# Successful clinical management of mesenteric ischemia caused by superior mesenteric artery obstruction and celiac artery stenosis: a case report

**DOI:** 10.11604/pamj.2023.44.18.35959

**Published:** 2023-01-09

**Authors:** Majed Mazen Fakeeh, Leena Hatem Moshref

**Affiliations:** 1 Department of Medicine, Dr. Soliman Fakeeh Hospital, Jeddah, Saudi Arabia,; 2 Department of Surgery, Dr. Soliman Fakeeh Hospital, Jeddah, Saudi Arabia

**Keywords:** Mesenteric ischemia, laparotomy, stents, case report

## Abstract

Acute mesenteric ischemia (AMI) is a rare, yet deadly thromboembolic accident that requires urgent surgery and or revascularization. We report the case of a 67-year-old male patient, who presented with severe abdominal pain and reduced oral intake, causing dehydration and impaired kidney function. The imaging evaluation including arterial Doppler and computed tomography (CT) scan showed AMI caused by superior mesenteric artery (SMA) obstruction and celiac artery stenosis, besides multiple atherosclerotic segments. Given the absence of guidelines in such an uncommon combination, a multidisciplinary management was initiated involving general medicine, general surgery, vascular surgery, and radiology. The agreed plan consisted of anticoagulation, exploratory laparotomy with necrosis resection and anastomosis, followed by percutaneous thrombectomy and angioplasty with stenting. The patient was discharged on day 7 postop with a highly satisfactory outcome and follow up. This case highlights the value of an early multidisciplinary approach in tailoring the management to the specific case of AMI.

## Introduction

Acute mesenteric ischemia (AMI) is an abrupt interruption of blood supply to a segment of the intestine leading to necrosis. Although the prevalence is low, accounting for 0.09-0.2% of all acute surgical admissions, AMI is associated with a high mortality reaching 50%, especially if untreated [1]. The diagnosis of AMI may be challenging due to unspecific clinical symptoms and inaccuracy of biological markers, while imaging, angiography, and endoscopy have a high diagnostic utility [1].

Patients with AMI necessitate immediate surgery, which should be combined, when possible, with endovascular revascularization for a better prognosis [2]. The initial management plan includes fluid resuscitation, antibiotic coverage, and therapeutic anticoagulation. Regarding surgical approach, although midline laparotomy is the golden standard, it is increasingly reserved for unstable patients with signs of peritonitis and unexplained sepsis [1]. Endarterectomy, bypass grafting, and mesenteric re-implantation are some of the open surgical revascularization procedures. However, the choice of the intervention may be determined by perioperative parameters including comorbidities, dietary state, and anatomical stability, as well as the setting´s expertise and resources [1,2].

We report the case of a 67-year-old patient with AMI caused by superior mesenteric artery (SMA) obstruction and celiac artery stenosis which was managed using a multidisciplinary approach.

## Patient and observation

**Patient Information:** a 67-year-old male patient with no surgical history presented to the gastroenterology clinic for a two-day postprandial pain associated with decreased oral intake. He passed gases and had no nausea or vomiting. Past medical history showed hypertension, heavy smoking, and old cerebrovascular accident on aspirin and clopidogrel.

**Clinical findings:** examination showed an alert, conscious, and well oriented patient. Vitals were unremarkable. The abdomen was soft and lax, with mild generalized tenderness and no rigidity. There was no lower limb oedema and other systems were unremarkable.

**Timeline of current episode:** two days before presentation (early July 2021): the patient complained of a severe abdominal postprandial pain associated with fear of eating due to anticipated pain.

**Figure 1:**
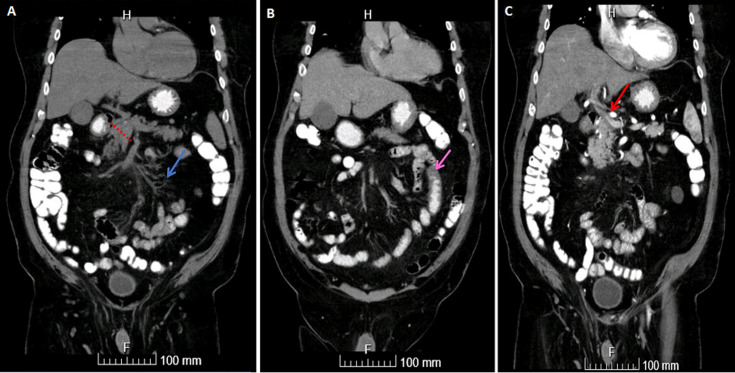
A,B,C) CT of abdomen and pelvis with contrast; the image showing occlusion in SMA (dashed line) and severe stenosis of its distal portion with occlusion of jejunal branches (blue arrow); a jejunal loop showing wall thickening (pink arrow) and submucosal oedema; stenosis of the celiac artery (red arrow)

**Diagnostic assessment:** presentation day: admission to internal medicine ward for suspicion of AMI. Blood investigations showed lactate 1.31 mmol/L, normal liver and pancreatic enzymes, besides elevated serum creatinine 1.37 mg/dl and an estimated glomerular filtration rate (eGFR) of 54 mL/min/1.73m^2^, likely secondary to dehydration and poor oral intake. An aortic Doppler was performed while an abdomen and pelvis CT scan with contrast was indicated but delayed because of impaired kidney function. The aortic Doppler showed atherosclerotic segments of the celiac trunk origin, and superior and inferior mesenteric arteries. Anticoagulation was started with enoxaparin 40 mg BID. Kidney function was normalized with IV fluids enabling the CT scan to be performed on the next day, which evidenced an occlusion of the superior mesenteric artery (SMA) and severe stenosis of its distal portion with occlusion of jejunal branches (Figure 1). A jejunal loop showed wall thickening and submucosal and surrounding oedema denoting ischemic enteritis, stenosis of the celiac artery, patent inferior mesenteric artery (IMA), and renal arteries, in addition to patent portal, superior mesenteric and splenic veins.

**Diagnosis:** findings were consistent with AMI.

**Figure 2:**
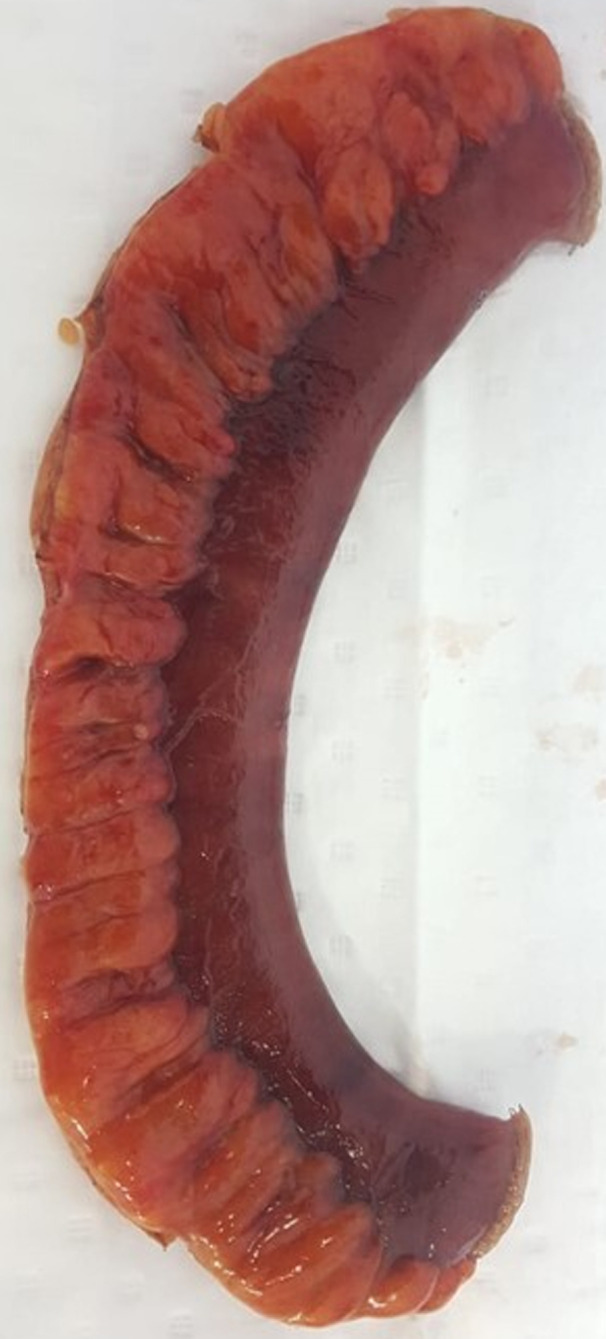
the resected segment that is congested and dusky

**Figure 3:**
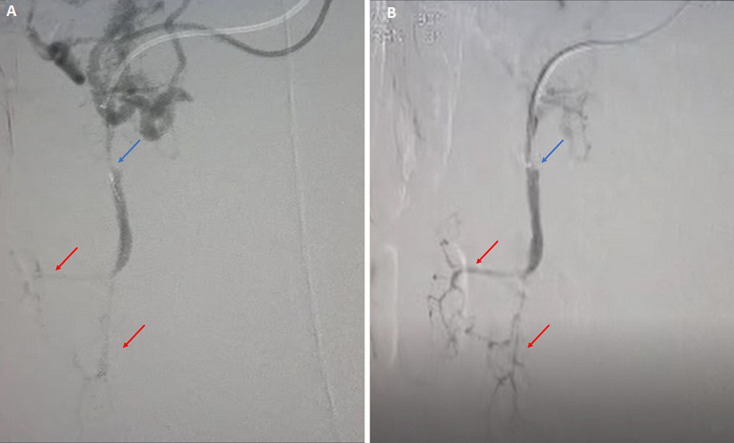
pre- and post-revascularization angiography showing angiography of the superior mesenteric artery (blue arrows) and jejunal branches (red arrows) before (A) and after (B) revascularization

**Therapeutic interventions:** a multidisciplinary meeting was held including a general surgeon, a vascular surgeon, and radiology team. A preoperative prophylaxis antibiotic therapy was started including cefuroxime 1500 mg IV and metronidazole 500 mg IV, and an exploratory laparotomy was performed, with bowel resection (Figure 2) and anastomosis. Pathology assessment of the resected small bowel loops showed ischemic changes, negative for granuloma, organisms, or malignancy. Proximal and distal resection margins are viable and free of ischemia. The patient was transferred to the vascular catheter laboratory and underwent SMA thrombectomy and angioplasty with stenting, via the left brachial artery. Angiography was performed, and peripheral findings showed stenosis concerning more than 90% of the celiac trunk distal and a mid SMA thrombosis. Final aortogram showed patent SMA down to iliac branches (Figure 3). Total fluoroscopy time was 31.23 mins and total fluoroscopy dose was 210780 mGycm^2^. Complications were none and blood loss was minimal.

**Follow up and outcome of interventions:** the patient was transferred to the critical care for observation. Day 1 postop: the patient was conscious with no complaints, and no vasopressor requirement. Enoxaparin dose was increased to 100 mg BID and clopidogrel 75 mg once daily was started. Day 2 postop: the patient was transferred to the regular surgical ward. On day 4 postop, he complained of moderate dyspnea, and examination showed bilateral basal crepitation and chest X-ray showed atelectasis. He was referred to a pulmonologist who recommended high-resolution computed tomography (HRCT) that showed mild bilateral pleural effusion and a focal atelectasis of the right middle lobe, with no signs of acute infection. Besides, HRCT showed coronary arterial atherosclerotic calcifications. He was started on chest physiotherapy and ipratropium and budesonide inhalation, in addition to chest physiotherapy every 6 hours and incentive spirometry. A cardiologist was consulted and started the patient on oral potassium chloride and bisoprolol 2.5 mg oral once daily. On day 7 postop, the patient was stable and was discharged home with a 6-month prescription of clopidogrel, apixaban, acetylsalicylic acid, and ezetimibe/atorvastatin. Additionally, the patient had metronidazole, cefuroxime, and pain killers, in addition to oral nutritional supplements with enhanced calories diet for 1 week. Furthermore, a regular follow up was scheduled on days 10-14 postop in four outpatient clinics including general surgery, cardiology, vascular surgery, and pulmonology. On cardiology follow up, a stress/rest myocardial perfusion imaging (MPI) was performed showing positive results, and a coronary artery bypass graft was planned. Vascular surgery follow up concluded to the continuation of dual anticoagulation (apixaban 5 mg BID and clopidogrel 75 mg) with follow up at 3 and for 6 months postop. On pulmonology follow up, additional inhalers were indicated after a trial of tiotropium4 inhalation, bilastine, and guaifenesin. Otherwise, the patient was asymptomatic.

**Patient perspective:** the patient expressed high satisfaction with the treatment and management plan. He appreciated and thanked all the medical teams.

**Informed consent:** it was obtained from the patient to publish this case.

## Discussion

The presented patient had an occlusion of the SMA with celiac artery stenosis, which represented a management challenge given the absence of guidelines and the rare combination. Acute embolism of the SMA is the most prevalent cause of enteric ischemia, accounting for 40 to 85% of all AMI cases [3]. The main origin of the embolism was reported to be arterial emboli from cardiac arrhythmias (40-50%) and thrombosis at preexisting lesions (25%), besides other non-occlusive sources. The SMA is the common site of the thromboembolism due to its oblique origin from the aorta [4]. However, depending on the diagnosis difficulty, the amount of bowel ischemia infarction, and the complexity of surgical revascularization, the perioperative mortality rate of thrombosis in the SMA may be very high [5]. This highlights the importance of keeping a high clinical suspicion even in atypical presentations.

In the present case, the clinical presentation was dominated by unproportionate abdominal pain that was described to be ‘unprecedented´ by the patient, whilst the physical examination was near-unremarkable. Severe abdominal pain that is disproportional to physical examination findings is one of the characteristics of AMI [2], while symptoms of peritonitis should suspect a permanent intestinal ischemia with colon necrosis [4]. Besides, although unspecific, laboratory data may aid to guide the diagnosis [1,5]. Leukocytosis is observed in more than 90% of the patients, and lactic acidosis is frequently found in case of dehydration and reduced oral intake [2]; however, none was observed in our case. In established AMI, a lactatemia >2 mmol/l is associated with permanent intestinal ischemia [2], and is associated with a high mortality [6]. Consequently, the combination of lactic acidosis with abdominal discomfort should prompt early computed tomography angiography (CTA).

Regarding management, there are no randomized controlled studies comparing laparotomy with endovascular therapy as a first-line treatment for AMI [1]. The ability to test bowel viability directly, thereby minimizing delays in restoring mesenteric blood flow, is the most compelling argument in favor of early laparotomy. For patients with overt peritonitis, it is highly advised (1A) to perform a timely laparotomy [1]. A retrospective study showed that one-third of patients benefitting from endovascular treatment avoided laparotomy [1]. Conversely, examining the intestinal function via laparoscopy may be a useful addition to endovascular approach [5].

Besides the absence of guidelines, there are a few cases that are similar to our case, which combined SMA obstruction and celiac artery stenosis. Therefore, the intervention scheme was decided based on a multidisciplinary conference comprising general surgery, vascular surgery, and a radiologist. Because the inflammatory indicators and lactic acid levels were within normal limits, there was some dispute on whether laparotomy should be followed by vascular intervention or vice versa. The risks and benefits were weighed, and the history of cerebral vascular accident (CVA) along with aspirin and clopidogrel medications were taken into consideration. It was decided to perform an exploratory laparotomy, to determine the viability of the bowel and anticipate bowel ischemia, followed by the vascular intervention. A study done by Chen *et al*. highlighted the advantages of combining laparotomy (direct bowel examination to establish viability) with endovascular intervention (prevent bypass grafting) in the same sitting, especially in individuals who otherwise require conventional surgical revascularization [5].

Given the associated celiac artery stenosis, the treatment plan was also determined based on the same multidisciplinary approach. It consisted of preoperative anticoagulation followed by resection of necrotic bowel and anastomosis, then percutaneous thrombectomy and angioplasty of the SMA with stenting. A case series from Brazil showed that embolectomy was performed in half of the patients, followed by mesenteric bypass and retrograde post-traumatic amnesia (PTA), while SMA stenting was performed in 13.3% of the cases [6]. Multiple percutaneous methods for AMI were described, among which aspiration and mechanical thrombectomy are the two most frequently used procedures [7]. A bypass graft from the left common iliac artery to the SMA was performed in a patient who had both celiac artery thrombosis and SMA stenoses [8]. In situations of refractory thrombus, balloon angioplasty is an alternate approach for clot breakup. Although there are a few published examples of percutaneous thrombectomy with or without PTA, the outcomes appear to be promising [7]. If performed early, angioplasty and stenting have been found to avoid intestinal infarction and eliminate the requirement for laparotomy [9].

Nevertheless, up to 70% of patients may still require laparotomy after recanalization of mesenteric arteries [3,10], and this has been associated with delay in diagnostic. Yet, it remains vital to consider endovascular surgery prior to abdominal surgery to enhance the intestine viability. A review of multiple meta-analyses found that endovascular surgery had a reduced prevalence of bowel resection and morbidity [2]. In more equipped settings, hybrid procedures, such as retrograde open mesenteric stenting (ROMS), showed promising results as they combine the benefits of open surgical and endovascular approaches [5].

## Conclusion

Acute mesenteric ischemia is a life-threatening condition that requires a high index of clinical suspicion and prompt diagnostic and therapeutic intervention. A highly-coordinated multidisciplinary management can enable establishing the diagnosis and tailoring the course of therapeutic action to the specific case of the patient.
